# Membrainy: a ‘smart’, unified membrane analysis tool

**DOI:** 10.1186/s13029-015-0033-7

**Published:** 2015-03-07

**Authors:** Matthew Carr, Cait E MacPhee

**Affiliations:** Institute for Condensed Matter and Complex Systems, School of Physics and Astronomy, The University of Edinburgh, Mayfield Road, Edinburgh, UK

**Keywords:** Molecular dynamics, Membrane analysis, Order parameters, Headgroup orientations, Mixing/Demixing entropy, Bilayer/Leaflet thickness, Area per lipid, Double bilayer, Asymmetric bilayer, Lipid flip-flopping

## Abstract

**Background:**

The study of biological membranes using Molecular Dynamics has become an increasingly popular means by which to investigate the interactions of proteins, peptides and potentials with lipid bilayers. These interactions often result in changes to the properties of the lipids which can modify the behaviour of the membrane. Membrainy is a unified membrane analysis tool that contains a broad spectrum of analytical techniques to enable: measurement of acyl chain order parameters; presentation of 2D surface and thickness maps; determination of lateral and axial headgroup orientations; measurement of bilayer and leaflet thickness; analysis of the annular shell surrounding membrane-embedded objects; quantification of gel percentage; time evolution of the transmembrane voltage; area per lipid calculations; and quantification of lipid mixing/demixing entropy.

**Results:**

Each analytical component within Membrainy has been tested on a variety of lipid bilayer systems and was found to be either comparable to or an improvement upon existing software. For the analytical techniques that have no direct comparable software, our results were confirmed with experimental data.

**Conclusions:**

Membrainy is a user-friendly, intelligent membrane analysis tool that automatically interprets a variety of input formats and force fields, is compatible with both single and double bilayers, and capable of handling asymmetric bilayers and lipid flip-flopping. Membrainy has been designed for ease of use, requiring no installation or configuration and minimal user-input to operate.

## Background

The cell membrane plays a crucial role in many biological systems, not only as a container for intracellular contents but also as an osmotic barrier, a platform for transmembrane proteins and fusion events, a means for maintenance of chemical and electrostatic potentials, and a barrier for drug and antibody delivery into the cell [1]. Cell membranes are primarily composed of phospholipids arranged in a bilayer and have been studied heavily with a host of experimental techniques including nuclear magnetic resonance, electron paramagnetic resonance and electron microscopy [2-5]. Recent developments in lipid force field parameters have lead to a wide range of molecular dynamics (MD) studies involving lipid bilayers which aim to improve the spatial and temporal resolution over existing experimental techniques [6]. In many of these studies, the membrane can be seen as a dynamic system that changes in response to environmental perturbations. Understanding the behaviour of the lipids can be crucial to understanding the behaviour of the system, e.g. to understand why certain amphipathic peptides prefer to insert into fluid or curved regions of the membrane [7,8], or to understand the effect a membrane-embedded object has on the mechanical properties of the surrounding lipids [9]. An improved understanding of the lipid behaviour in response to environmental perturbations may lead to advances such as improved drug design and delivery into the cell [10].

There are several tools freely available to analyse individual components of the membrane such as its thickness, curvature, area per lipid, or acyl chain order parameters [11-13]; however, we were unable to locate tools that provide measurements of other membrane properties such as headgroup orientations, gel/fluid ratios, lipid mixing/demixing entropy, etc. Furthermore, many of these existing tools are targeted towards the experienced user, requiring complicated installations and configuration files to operate. Existing platforms for the creation of analytical tools such as MDTraj [14] and MDAnalysis [15] rely on the installation of additional packages to operate and as such may be problematic for non-Linux users.

We present Membrainy, an intelligent membrane analysis tool that endeavours to provide both the inexperienced and experienced user access to a wide range of analytical techniques to enable the measurement of various membrane-specific properties from planar bilayer trajectories. Membrainy was designed for simplicity and ease of use, requiring no compilation and minimal user-input to operate. As the range of lipid bilayer studies is broad, Membrainy was designed to automatically interpret a variety of bilayer compositions and force fields, and is capable of interpreting single, double and asymmetric bilayers. Membrainy can interpret dynamic membranes that undergo structural changes such as lipid flip-flopping, and employs different analytical approaches when switching between atomistic, united-atom or coarse grained force fields. A suite of analytical techniques is integrated within Membrainy. Acyl chain order parameters quantify the degree of order in the lipid tails, a measure often associated with lipid fluidity [16-18]. Headgroup orientations provide a measurement of the angles observed in the lipid headgroup relative to the membrane surface, and have been shown to be sensitive to electric charges and dipole fields [19]. Lipid mixing/demixing entropy is a quantification of the level of mixing between two or more lipid types, which plays an important role in a wide variety of cellular functions including DNA fusion and phase transitions [20]. The transmembrane voltage (TMV) across a double bilayer can be measured over time, and may be of particular importance in electrophysiology or electroporation simulations [21-23]. Generation of surface maps provide a high resolution 2D representation of the bilayer surface and is particularly helpful when looking at defects, undulations and gel clusters that may not be easily observable in 3D visualisation software. Gel percentages quantify the fluidity of the bilayer by measuring the linearity of the lipid tails. Measurements of leaflet and membrane thickness may be of importance in simulations where bilayers undergo electrostriction [24]. The detection and measurement of lipid flip-flopping may be useful in bilayers containing transient water pores [25]. Finally, the ability to perform a separate analysis on the annular shell of lipids surrounding molecules, whether inserted or in close contact with the membrane surface, may be helpful in understanding how these molecules affect the local properties of the membrane, such as changes in lipid tail flexibility [26].

## Implementation

Membrainy has been written in Java, which provides maximum compatibility across a range of operating systems, requires no compilation and enables the safe and efficient execution of multithreaded code. Membrainy contains various multithreaded algorithms to optimise efficiency and processor use across a range of architectures. These include algorithms for using multiple threads to load larger trajectory files, for preloading the next frame in the trajectory while the current frame is being analysed, and for running each analytical technique in parallel. Membrainy has been primarily designed for use with the GROMACS MD package [13], and contains a user interface that should be intuitive to GROMACS users. Membrainy is capable of reading GROMACS xtc, trr, tpr, cpt and gro trajectory file types, along with the standard pdb trajectory file type used by other MD packages (e.g. AMBER [27], CHARMM [28], NAMD [29], etc.). Membrainy has been implemented with the CHARMM36 [30], Berger/GROMOS87 [31] and Martini v2.0 [32] force fields, and is expandable to include other force fields and trajectory formats. Asymmetric bilayers and lipid flip-flops are detected by assigning each lipid to a corresponding leaflet depending on the height of its phosphorous atom relative to the geometric centre of the bilayer. All output graphs are readable by the Grace plotting software [33] and are preprogrammed with appropriate axis labels and titles. Double bilayer systems are automatically detected and incur additional output plots which contain averages of the inner and outer leaflets for certain analytical techniques.

### Order parameters

Order parameters for saturated and unsaturated lipid tails in atomistic force fields are calculated from the equation

(1) $$ S_{CD} = \left \langle \frac{3cos^{2}\theta - 1}{2} \right \rangle   $$

where *θ* is the angle the C −H bond vectors along the lipid tails make with the membrane normal [34], taken as the *z*-axis for planar bilayers. This approach utilises each individual C −H bond in the lipid tails. As united-atom force fields lack non-polar hydrogen atoms, the above equation is modified to include the relation

(2) $$ S_{CD} = \frac{2}{3}S_{xx} + \frac{1}{3}S_{yy}  $$

which is derived from the order parameter tensor [35], and achieved by defining molecular axes where the *z*-axis encompasses the C _*i*−1_−*C*_*i*+1_ vector, the *y*-axis lies on the plane containing C _*i*−1_−*C*_*i*_−*C*_*i*+1_, and the *x*-axis is orthogonal to the *y* and *z* axes. The angles that the *x* and *y* axes make with the membrane normal is then used to determine *S*_*xx*_ and *S*_*yy*_ from Equation 1. Martini order parameters are calculated from the equation

(3) $$ P_{2} = \frac{1}{2} \left (3\:cos^{2} \left \langle \theta \right \rangle -1 \right)  $$

where *θ* is the angle between the lipid tail bonds and the membrane normal.

The final order parameter for each technique is averaged over all leaflets in the system, and Membrainy will also produce separate order parameters for each lipid type and leaflet. For atomistic and united-atom force fields, Membrainy plots the values of −*S*_*CD*_ for each carbon along the lipid tails. This experiences maximum order at 0.5 and disorder at -1, whereas the Martini force field experiences maximum order at *P*_2_=1 and disorder at *P*_2_=−0.5. Membrainy can also produce histograms of the angles measured by each technique. To maximise performance, the order parameter algorithms are multithreaded, where each lipid tail type (e.g. POPE-palmitoyl, POPE-oleoyl, etc.) is assigned its own thread, allowing much of the analysis to be conducted in parallel.

### Headgroup orientations

Membrainy calculates lateral and axial headgroup orientations, producing a histogram for each lipid type. The lateral angles are calculated by establishing a headgroup vector from two reference atoms, one being the phosphorous atom and the other being another atom on the headgroup. This vector is then projected onto the membrane normal to produce an angle. The histograms are plotted in the range -90 to 90 degrees, where a value of 0 indicates the headgroup is parallel to the membrane surface and positive angles indicate the headgroup is pointing away from the membrane. Axial angles are calculated by projecting the headgroup vector onto the membrane surface, taken as the *xy* plane, to produce a radial angle between 0 and 2 *π*. Each axial angle is plotted for each lipid over time. This algorithm has been multithreaded, where each lipid type is assigned its own thread and run in parallel.

### 2D surface maps

The membrane surface can be represented in a 2D map by binning the heights of each atom in each leaflet into a 2D lattice and applying the Gauss-Seidel method

(4) $$ \phi_{i,j}^{n+1} = -\frac{1}{4}\left [ A_{i,j} - \left (\phi_{i-1,j}^{n} + \phi_{i+1,j}^{n} + \phi_{i,j-1}^{n} + \phi_{i,j+1}^{n} \right)\right ]  $$

where *A*_*i*,*j*_ is the highest atom in cell *i*,*j*, $\phi _{i,j}^{n+1}$ is the resulting scalar value produced by the method, and the final term is the sum of the neighbouring cells’ scalar values. Iterating over this method produces a scalar field of successive displacement, generating a series of Gaussians that can be scaled and mapped to a colour to produce a contour map of the leaflet surface. These maps also behave as density maps, producing more prominent Gaussians in regions of the lattice containing a high density of atoms, such as lipid tails in the gel phase. The scalar field is colour-coded such that blue regions indicate thin or sparsely populated regions of the leaflet, red indicates thick or densely populated regions, with green between the two. Black areas represent a hole or pore in the leaflet, which is identified by unpopulated regions of the lattice. A map for each leaflet is displayed through a graphical interface in real-time and can be saved as an image. Membrainy will also overlay the positions of molecules and ions on the maps. As iterative approaches can be computationally expensive, each leaflet is assigned its own thread allowing the maps to be generated in parallel.

### Leaflet/membrane thickness, area per lipid and gel percentage

Membrane thickness is determined by calculating the average height of a user-specified reference atom, typically the phosphorous atom, for each leaflet. The average height of the reference atom for two opposing leaflets can then be subtracted. Leaflet thickness is calculated by subtracting the average height of the reference atom with the geometric centre of the bilayer. A 2D thickness map can also be produced by binning the reference atoms into a 2D lattice and applying the same algorithm used by the 2D surface maps. Membrainy offers a simple area per lipid (APL) calculation by dividing the box area by the number of lipids per leaflet, and will automatically produce multiple APLs for asymmetric bilayers or when lipid flip-flopping is detected. Gel percentages can be approximated by comparing the force field distance between the first and last carbon atoms in the lipid tails with the distance found in the trajectory files. As fluid lipid tails are non-linear, this distance is typically much less than the force field distance. A user-specified tolerance is assigned to the force field distance, and any lipid with a trajectory distance above this tolerance is counted as a ‘gel’ lipid.

### Annular shell analysis

Membrainy isolates the annular shell of lipids around molecules by calculating a distance vector between each atom in the bilayer with each atom in the molecule. If the distance between any two atoms is within a user-specified radius, the lipid is counted as being within the shell. These lipids can then be analysed to determine their properties. A control group can also be established by selecting random lipids outside of the shell from the same leaflet, comprising either a fixed number of lipids, an identical number of lipids to those found within the shell or all lipids outside of the shell. An option exists to exclude gel lipids from the control group, as many proteins and peptides are known to show selectivity for inserting into fluid regions [36]. Gel lipids are identified using the same technique described above. If multiple molecules are present, the user may specify one, several or all molecules to construct annular shells for, and Membrainy will assign a thread to each molecule, populating the shells in parallel. The output plots contain an average of all shells in the system. Membrainy is also fitted with an annular shell analysis algorithm to produce detailed records of which lipids occupy the shell at any given time and which lipids spent the longest time in the shell. In mixed bilayer compositions, Membrainy will plot the ratio of lipid types found within the shell over time.

### Evolution of the TMV

In double bilayer systems, the TMV can be extrapolated from the average electrostatic potential between the two bilayers, which is calculated from a double integral of Poisson’s equation

(5) $$ \Psi (z) = - \frac{1}{\varepsilon_{0}}{\int_{0}^{z}}dz^{\prime} \int_{0}^{z^{\prime}} \rho \left(z^{\prime\prime}\right)dz^{\prime\prime}   $$

and is achieved by splitting the simulation box into ‘slices’ along the *z*-axis and calculating the charge density in each slice [37]. The box is then corrected such that *Ψ*(0)=0. Membrainy utilises the GROMACS tool g_potential by splitting the full trajectory into smaller trajectories and calculating the electrostatic potential in each trajectory. The TMV can then be extrapolated from each smaller trajectory and recombined to produce a voltage against time measurement over the full trajectory.

### Lipid mixing/demixing entropy

Membranes containing two or more lipid types can have their lipid mixing/demixing quantified as an entropy with the equation

(6) $$ S(x_{1},\!..,x_{N}) = N \sum\limits_{x_{i},nb_{i}} p(x_{i},nb_{i})\: log\: p(x_{i}\mid nb_{i})   $$

as described by Brandani et al. [38], where *p*(*x*_*i*_,*nb*_*i*_) is the probability to find a lipid of type *x*_*i*_ neighboured to a lipid of type *nb*_*i*_, and *p*(*x*_*i*_∣*nb*_*i*_) indicates the conditional probability that a lipid is of type *x*_*i*_ given that its neighbour is of type *nb*_*i*_. To calculate the entropy, a distance vector is established between the phosphorous atoms on each lipid in a leaflet to determine the nearest neighbouring lipid and its type. This information is then binned into a probability matrix and normalised such that the total probability is always 1, and then used with Equation 6 to produce an entropy. A theoretical maximum entropy can be calculated from

(7) $$ S_{max} = -\sum \rho_{x_{i}} \: log \: \rho_{x_{i}}  $$

where $\rho _{x_{i}}$ is the density of a lipid of type *x*_*i*_. A scaled entropy is also produced such that *S*_*max*_=1.

## Results and discussion

Membrainy was tested on Linux 64-bit machines containing 2-8 cores on a selection of single and double bilayer trajectories employing the CHARMM, GROMOS and Martini force fields. Where appropriate, results were compared with either existing software, experimental values, or judged for logical consistency.

### Order parameters

Order parameters are a measure of the level of order or entropy in the lipid tails and can give insight into the fluidity of the membrane, as gel lipids exhibit a greater degree of order over fluid lipids. Membrainy was used to generate order parameters for various bilayer compositions, which found saturated lipid tails to be comparable to those produced by the GROMACS tool g_order in the CHARMM and GROMOS force fields. For bilayers employing the CHARMM force field, Membrainy yields more accurate order parameters to g_order by utilising each C −H bond vector, whereas g_order ignores these vectors and instead reconstructs them from the C _*i*−1_−*C*_*i*+1_ vector in a similar approach used by Membrainy with united-atom force fields. The order parameters for unsaturated lipids were also comparable, excluding the region around the double bond in which g_order calculates incorrectly. Membrainy automatically generates the necessary lipid tail information required to calculate order parameters, requiring no user-input. This is a significant improvement over g_order which requires a lengthy setup of user-constructed index files. This also restricts the order parameter analysis to a fixed number of lipids and as such it would be difficult to conduct an annular shell analysis with g_order.

### Annular shell analysis

**Figure 1:**
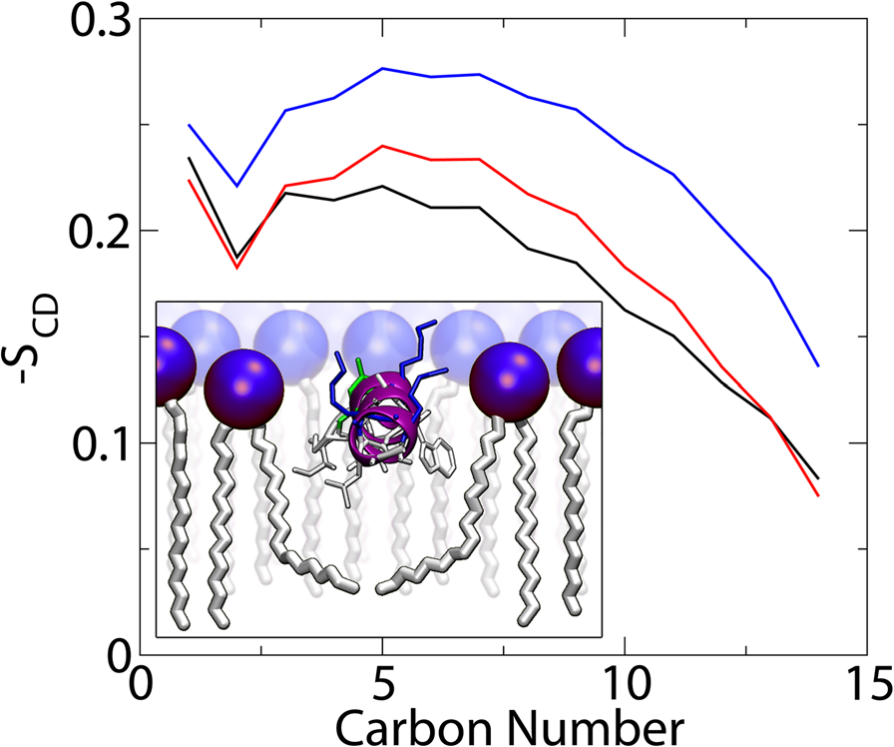
**Annular shell order parameters.** The order parameters of saturated lipid tails from an annular shell analysis of MinD-MTS, an amphipathic helical peptide inserted into a POPE/POPG (3:1) double bilayer at 300K. The shell order parameters are shown in black, along with two control groups: the red plot uses the option built into Membrainy to ignore all gel lipids, which produces a more accurate control group for this peptide as it resides in a fluid region of the bilayer; and the blue plot contains both gel and fluid lipids. The differences between the black and red plots indicate the presence of splayed lipid tails in the annular shell, whereas the blue plot is sampling the wrong phase of lipids and provides an inaccurate comparison to the lipids within the annular shell.

The properties of the lipids in close proximity to other molecules, whether inserted or in close contact with the membrane surface, may be modified by the presence of such molecules and result in local changes to the membrane. Membrainy isolates an annular shell of lipids surrounding a peptide, protein or other molecule, and compares its properties to lipids outside of the shell. As an exemplar, we inserted the MinD membrane targeting sequence (MinD-MTS) into the headgroup region of a POPE/POPG (3:1) double bilayer. This peptide is an 11-residue cationic amphipathic helix located at the C-terminus of the MinD protein that plays an important role in the cell division of *Bacillus subtilis* [39]. Using a shell radius of 4 Å, the order parameters of the lipids located within the shell were analysed over 50 ns and compared with a control group, comprising an identical number of lipids selected randomly from outside of the shell within the same leaflet. The order parameters reveal an increase in disorder for lipid tails within the shell when compared to those outside of the shell (Figure 1), which suggests the presence of splayed lipid tails. This phenomenon has previously been predicted for amphipathic peptides inserted into the headgroup region of lipid bilayers [26]. The option to ignore all gel lipids from the control group was enabled as our chosen peptide had inserted into a fluid region of the bilayer that contained ∼27% gel at 300 K. Without this option, the control group was observed to sample a more ordered phase of lipids, providing an inaccurate comparison with the fluid lipids found within the shell. The annular shell analysis algorithm revealed that lipids were continuously entering and exiting the shell, and saw approximately 10-14 lipids occupy the shell at any given time. Membrainy can also determine lipid type ratios within the shell for mixed bilayer compositions, which may be useful when studying molecules that give rise to an enrichment of certain lipid types. This measurement revealed an average lipid ratio of 2.6:1 POPE:POPG within the shell, suggesting that MinD-MTS gives rise to an enrichment of POPG lipids, likely mediated by the increased electrostatics between the cationic peptide and anionic POPG headgroups.

### Evolution of the TMV

**Figure 2:**
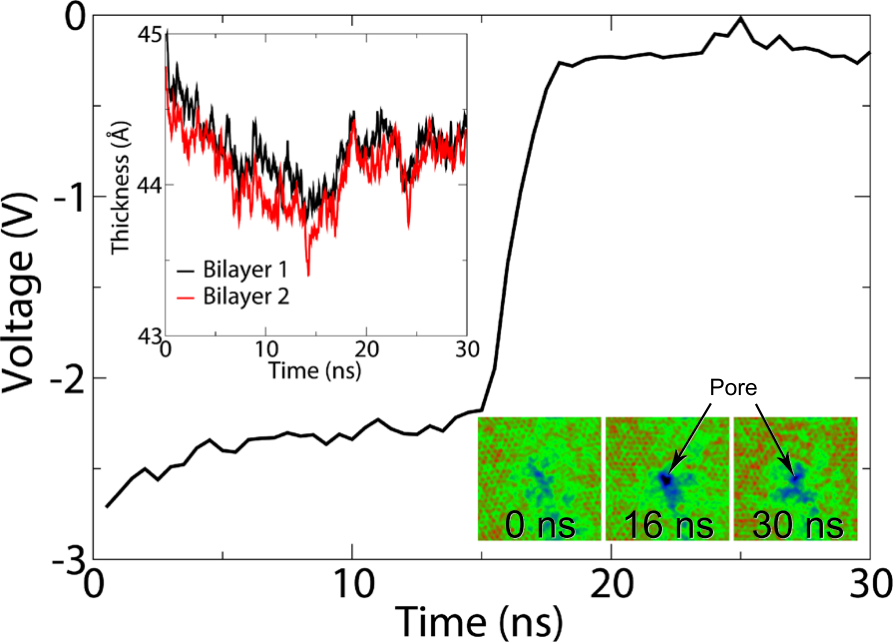
**Evolution of the TMV and membrane thickness.** A POPE/POPG (3:1) double bilayer was subject to an ion imbalance of +20, achieving an initial TMV of -2.65 V. Within 5 ns, the TMV lowers to -2.35 V as the bilayers expand laterally and experience a thickness reduction due to electrostriction. At 15 ns, a transient water pore formed through electroporation, allowing ions to travel through the pore in opposite directions. This resulted in a rapid loss of the initial ion imbalance which incurs a sharp drop in TMV. By 17 ns, the TMV is insufficient to maintain electrostriction, allowing the bilayer thickness to increase.

The TMV is the electrical potential found across biological cell membranes and plays a crucial role in a wide range of cellular processes, including the transport of nutrients into and out of the cell, biophysical signaling, and cell proliferation [40-42]. Membrainy is capable of measuring the time evolution of the TMV across a double bilayer, which may be of importance in electrophysiology and electroporation simulations. As an exemplar, numerous electroporation simulations were conducted for 30 ns using POPE/POPG (3:1) double bilayers. These systems were initially established with ion imbalances of +20, achieved by moving 10 cations from the inner (anodic) water compartment to the outer (cathodic) water compartment, similar to the approach taken by Sachs et al. [43]. Transient water pores were observed to form after a random time interval, allowing both cations and anions to travel through the pores in opposite directions, resulting in a loss of the initial ion imbalance. Membrainy was used to produce TMV against time measurements for each simulation, one of which is depicted in Figure 2. This measurement reveals an initial TMV of -2.65 V, which lowers to -2.35 V during the first 5 ns due to the lateral expansion of the bilayers undergoing electrostriction. Once a pore had formed, a sharp drop in TMV is observed at a rate of 0.75 V/ns, corresponding to ion transport through the pore at a rate of 3 ions/ns. The resulting TMV is indicative of the remaining ion imbalance (+2). These measurements provide an informative way to monitor changes to the TMV during a trajectory, and can be used in electroporation simulations to determine the time at which a pore is formed and the rate at which the TMV is dissipated.

### Leaflet/membrane thickness and area per lipid

Using the same electroporation simulation as above, Membrainy was used to measure the leaflet and membrane thickness over the trajectory (with the membrane thickness depicted in Figure 2). This membrane thickness steadily decreases prior to pore formation, corresponding to the electrostriction effects experienced by the bilayers from the TMV. After pore formation, the bilayer thickness sharply increases as the TMV is dissipated, suggesting that the electrostriction effects had diminished and the bilayers were able to relax towards their initial thickness. Similar results were obtained for the leaflet thickness, and interestingly the anodic leaflets were observed to be consistently thinner than the cathodic leaflets prior to pore formation, which was also observed by Böckmann et al. in a similar electroporation study [44]. Membrainy was used to calculate the APL during the simulation, which revealed an increase in APL prior to pore formation, and a decrease afterwards. This result was identical to the APL produced with the GROMACS tool g_energy, which can output the box dimensions over time to be converted to an APL.

### Lipid flip-flopping

Transmembrane lipid translocation, more commonly known as lipid flip-flopping, is the process in which lipids are translocated between the two opposing leaflets of a bilayer [45]. This translocation occurs from both passive and active transport mechanisms and plays a crucial role in the maintenance of asymmetric cell membranes [46]. Lipid flip-flopping has also been observed in simulated DMPC bilayers through electroporation, whereby lipids translocate through transient water pores in both directions [25].

**Figure 3:**
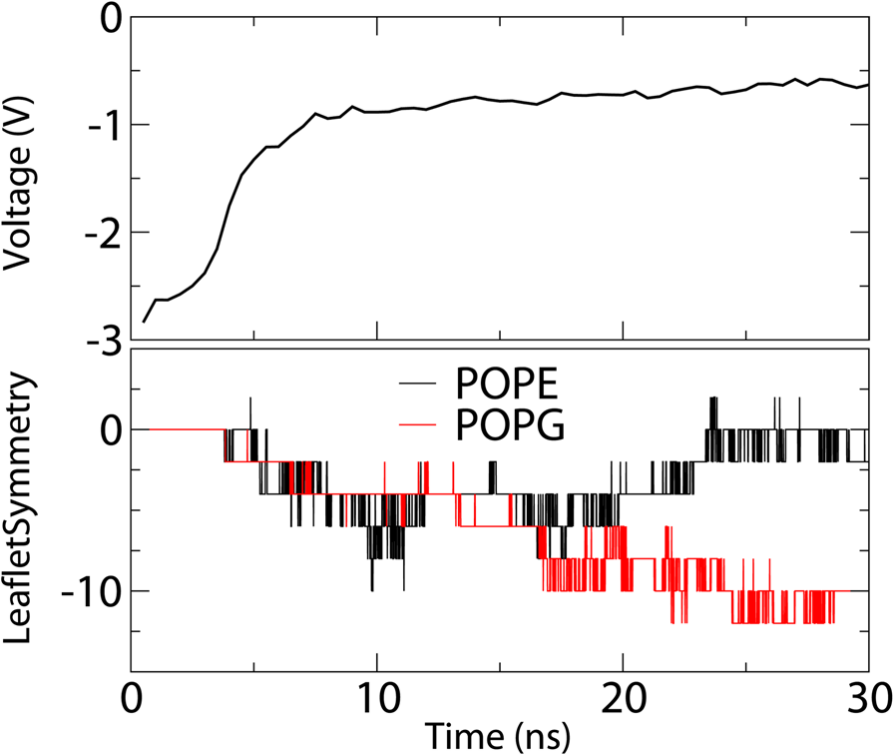
**Lipid flip-flopping.** TMV and leaflet symmetry measurements of a POPE/POPG (3:1) double bilayer undergoing electroporation over 30 ns. A value of -2 in leaflet symmetry indicates a single flip-flop from the anodic to the cathodic leaflet. A pore was formed within 5 ns, which saw both POPE and POPG lipids from the anodic leaflet form the toroidal structure of the pore. After 15 ns, the POPE lipids within the pore return to the anodic leaflet while additional POPG lipids translocate to the cathodic leaflet. By 30 ns, one POPE lipid had flip-flopped from both leaflets (producing a symmetry of 0) and five POPG lipids had flip-flopped to the cathodic leaflet. This suggests that POPG lipids are more susceptible to flip-flopping towards the cathodic leaflet through transient water pores when under the influence of a TMV.

Membrainy was used to detect lipid flip-flopping during a 30 ns simulation of a POPE/POPG (3:1) double bilayer, in which an ion imbalance of +20 was maintained with position restraints. A pore formed within 5 ns and remained open for the duration of the simulation. Figure 3 depicts the TMV and leaflet symmetry measurements during the simulation, where the leaflet symmetry is calculated by subtracting the number of lipids in the cathodic leaflets from the number of lipids in the anodic leaflets, and therefore a value of -2 indicates a single flip-flop to the cathodic leaflet. Upon pore formation, the leaflet symmetry reveals that the toroidal structure of the pore mainly comprised POPE and POPG lipids from the anodic leaflet. This is likely due to the tendency for transient water pores to initiate formation from the anodic water compartments, as observed by Böckmann et al. [44]. After 15 ns, the POPE symmetry returns to zero, indicating that the distribution of POPE lipids across both leaflets has equalised; however, the POPG symmetry steadily decreases, indicating that POPG lipids are translocating through the pore towards the cathodic leaflet. By 30 ns, one POPE lipid in each leaflet had flip-flopped and five POPG lipids had flip-flopped to the cathodic leaflet. This suggests that POPG lipids experience a greater tendency to flip-flop through transient water pores in bilayers subject to a high voltage TMV, in which the POPG lipids are translocated towards the cathodic leaflet, likely due to the additional forces acting on the anionic POPG headgroups from the electric field. Membrainy has therefore detected and interpreted lipid flip-flopping through a transient pore within this system.

### 2D surface maps and gel percentage

**Figure 4:**
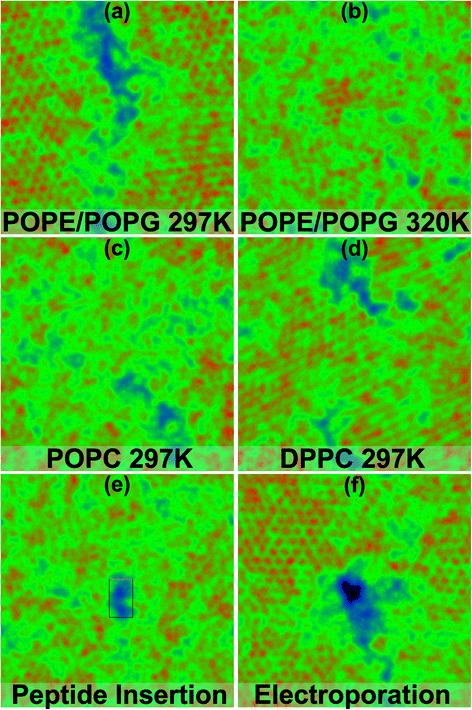
**2D surface maps.** These maps depict leaflets taken from a variety of lipid bilayer simulations. Red hexagonally packed dots represents gel clusters and black areas indicate the presence of a pore or hole in the leaflet. **(a)** and **(b)** depict POPE/POPG (3:1) bilayers at two temperatures, where (a) is near the transition temperature and contains ∼53% gel, and (b) is in the fluid phase and contains ∼14% gel. **(c)** and **(d)** depict POPC and DPPC bilayers at 297 K, containing ∼16% and ∼85% gel respectively. These percentages correspond with the correct phase of each bilayer as 297 K is above the transition temperature for POPC and below that of DPPC. The DPPC map also reveals a smudged appearance to the gel clusters which is indicative of lipids in the tilted $L_{\beta ^{\prime }}$ phase. **(e)** depicts an inserted MinD-MTS peptide in a POPE/POPG (3:1) bilayer at 300 K. **(f)** depicts a leaflet containing a transient water pore established through electroporation in a POPE/POPG (3:1) bilayer at 297 K.

Surface maps were generated for POPE/POPG (3:1) bilayers at 297 K, 300 K and 320 K, and DPPC and POPC bilayers at 297 K using the CHARMM force field (Figure 4). The 300 K POPE/POPG bilayer contained the MinD-MTS inserted into the headgroup region, and the 297 K POPE/POPG bilayer was imaged before and during electroporation. As 297 K is approximately the transition temperature for POPE/POPG bilayers [47], Membrainy detected ∼53% gel within the bilayer. This percentage is accurately portrayed in the surface map (Figure 4a) where approximately half of the map appears as gel, represented by hexagonally packed red dots (the hexagonal packing of lipid tails occurs naturally in gel domains). The same bilayer was analysed at 320 K and showed ∼14% gel, which is also portrayed in the surface map (Figure 4b) by showing fewer gel clusters. The POPC bilayer at 297 K shows a highly fluid bilayer with ∼16% gel (Figure 4c), whereas the DPPC bilayer at 297 K shows ∼85% gel (Figure 4d). These measurements are in agreement with their corresponding transition temperatures of 271 K for POPC bilayers and 314 K for DPPC bilayers [48]. Interestingly, the DPPC bilayer exhibits gel lipids in the tilted $\phantom {\dot {i}\!}L_{\beta ^{\prime }}$ phase which is portrayed in the surface map by the smudged appearance of the gel clusters. Finally, surface maps were generated for the bilayer containing an inserted MinD-MTS peptide (Figure 4e) and the bilayer from the previous electroporation simulation containing a transient water pore (Figure 4f). These 2D surface maps provide an alternative representation of the bilayer, capable of producing both a contour and density map that portrays gel clusters, pores, surface undulations and defects that may not be easily seen in 3D visualisation software such as VMD [49] and Pymol [50].

### Headgroup orientation

The lipid headgroup is the polar interface between the membrane core and the intracellular/extracellular spaces and has been observed to exhibit sensitivity to electric charges, dipole fields, and temperature effects [51,52]. Experimental techniques have shown the lipid headgroup to sit roughly perpendicular to the lipid tails with a variation of around 30 degrees to the membrane surface [19,53]. In MD simulations, measurements of lipid headgroup orientations can provide an effective means to compare bilayers undergoing environmental perturbations, such as those under the influence of a TMV. As an exemplar, a POPC double bilayer was equilibrated for 100 ns without a TMV, after which a 30 ns simulation was conducted using an ion imbalance of +28, achieving a TMV of -1.95 V. Membrainy was used to measure the headgroup orientations before and after applying a TMV, which yielded a mean angle of 23 degrees in both leaflets without a TMV, and mean angles of 25.5 and 21.5 degrees in the anodic and cathodic leaflets respectively after applying a TMV. This reveals a shift of +2.5 degrees in the anodic leaflets and -1.5 degrees in the cathodic leaflets, suggesting that the headgroups in both leaflets are tending to align with the electric field. These measurements are comparable to those found by Böckmann et al. [44].

### Lipid mixing/demixing entropy

**Figure 5:**
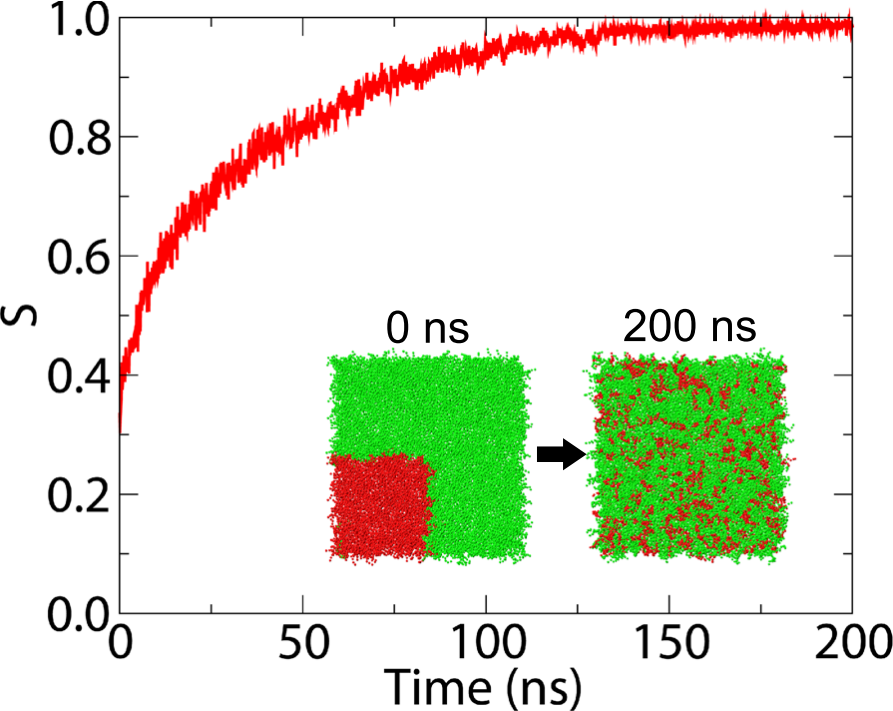
**Mixing entropy.** The mixing entropy of a POPE/POPC (3:1) bilayer over 200 ns, scaled such that *S*_*max*_=1. The bilayer is initialised such that POPC lipids (shown in red) encompass the lower left quadrant of the bilayer and the remaining bilayer contains POPE lipids (shown in green), creating a perfectly demixed system. An initial entropy of 0.3 is observed, which increases as the lipid types mix together. By 150 ns, the resulting entropy settles just below the theoretical maximum entropy, indicating a perfectly mixed system.

The entropy of lipid mixing/demixing provides a measure of the two-dimensional spatial heterogeneity of any lipid bilayer system, and a means to study changes following an environmental perturbation. A bilayer was constructed containing 1512 POPE and 504 POPC Martini lipids, where the POPC lipids were initially clustered together in a quadrant of the bilayer creating a perfectly demixed system. This bilayer was simulated for 200 ns and its trajectory was analysed by Membrainy to produce a scaled mixing entropy over time (Figure 5). These measurements reveal an initial entropy of 0.3, which immediately increases as the system began to mix. By ∼150 ns the entropy settles just below the theoretical maximum entropy, indicating the bilayer was fully mixed. Membrainy has therefore quantified the level of mixing/demixing in this system.

## Conclusions

Membrainy is an important tool for any membrane simulation where the lipids may undergo changes in response to environmental perturbations. Membrainy was designed to be simple and powerful, requiring no compilation and minimal user-input to run, and offers a wide range of analytical techniques for the calculation of various membrane-specific properties including those that, to our knowledge, are not currently available with existing software. Membrainy is capable of automatically interpreting a wide range of lipid bilayer systems, including those with complex lipid compositions, or those utilising single, double or asymmetric bilayers. Membrainy will automatically detect the force field in use, and is able to adapt to dynamic membranes that undergo structural changes such as lipid flip-flopping. We have shown Membrainy to be a useful and effective tool for analysing a broad scope of biological effects and environmental perturbations acting on lipid bilayers that may incur changes to the lipids and therefore modify the properties of the membrane.

## Availability and requirements

**Project Name:** Membrainy **Project home page:**www.membrainy.net**Operating systems:** Platform independent **Programming language:** Java **Other requirements:** Java v1.6 or higher, GROMACS v4 or higher (to enable some features) **License:** GNU GPL v2 **Any restrictions to use by non-academics:** None

## Abbreviations

APL: Area per lipid
MD: Molecular dynamics
MinD-MTS: MinD membrane targeting sequence
POPC: 1-palmitoyl-2-oleoyl-sn-glycero-3-phosphocholine
POPE: 1-palmitoyl-2-oleoyl-sn-glycero-3-phosphoethanolamine
POPG: 1-palmitoyl-2-oleoyl-sn-glycero-3-phosphoglycerol
TMV: Transmembrane voltage
